# Denoising Autoencoder Normalization for Large-Scale Untargeted Metabolomics by Gas Chromatography–Mass Spectrometry

**DOI:** 10.3390/metabo13080944

**Published:** 2023-08-13

**Authors:** Ying Zhang, Sili Fan, Gert Wohlgemuth, Oliver Fiehn

**Affiliations:** West Coast Metabolomics Center, UC Davis, 451 Health Sciences Drive, Davis, CA 95616, USA; yzhang1088@gmail.com (Y.Z.); slfan@ucdavis.edu (S.F.); wohlgemuth@ucdavis.edu (G.W.)

**Keywords:** GC–MS, data normalization, statistics, primary metabolism, derivatization

## Abstract

Large-scale metabolomics assays are widely used in epidemiology for biomarker discovery and risk assessments. However, systematic errors introduced by instrumental signal drifting pose a big challenge in large-scale assays, especially for derivatization-based gas chromatography–mass spectrometry (GC–MS). Here, we compare the results of different normalization methods for a study with more than 4000 human plasma samples involved in a type 2 diabetes cohort study, in addition to 413 pooled quality control (QC) samples, 413 commercial pooled plasma samples, and a set of 25 stable isotope-labeled internal standards used for every sample. Data acquisition was conducted across 1.2 years, including seven column changes. In total, 413 pooled QC (training) and 413 BioIVT samples (validation) were used for normalization comparisons. Surprisingly, neither internal standards nor sum-based normalizations yielded median precision of less than 30% across all 563 metabolite annotations. While the machine-learning-based SERRF algorithm gave 19% median precision based on the pooled quality control samples, external cross-validation with BioIVT plasma pools yielded a median 34% relative standard deviation (RSD). We developed a new method: systematic error reduction by denoising autoencoder (SERDA). SERDA lowered the median standard deviations of the training QC samples down to 16% RSD, yielding an overall error of 19% RSD when applied to the independent BioIVT validation QC samples. This is the largest study on GC–MS metabolomics ever reported, demonstrating that technical errors can be normalized and handled effectively for this assay. SERDA was further validated on two additional large-scale GC–MS-based human plasma metabolomics studies, confirming the superior performance of SERDA over SERRF or sum normalizations.

## 1. Introduction

Metabolome is defined as the complete set of low-molecular-mass compounds (<1500 Da) synthesized or modified by a living cell or organism. Metabolomics is the simultaneous measurement of all small molecular metabolites that participate as substrates, reactants, signaling agents, intermediates, and products of enzyme-mediated reactions [1]. Mass spectrometry-based metabolomics has matured as a high-throughput, high-resolution, and high-dimensional technique that identifies multiple metabolite markers present in significantly different abundances between different conditions in large human cohorts or other biomedical and biological studies, enabling the discovery of diagnostic and predictive metabolite levels for disease [2,3].

Metabolomics can be integrated with transcriptomics and proteomics to find biomarkers of diseases or to elucidate biological mechanisms. For both goals, high-quality data mining is needed that removes unwanted (technical) variance. Such technical variance is impacted by various forms of unwanted variations in conducting laboratory experiments, from batch-to-batch differences, variation between different instruments, inter-person variation, and drifts in instrument sensitivity across a specific sequence of samples [4]. To extract the biologically relevant information, such technical variance needs to be efficiently removed via data normalization methods after raw-data acquisition. Classic quantification strategies in analytical chemistry employ exogenous chemical surrogates as quality controls and for normalization against matrix effects, using either stable isotope-labeled chemicals (deuterium or ^13^C labeled) or structural analogs of target molecules. Because metabolomics aims to analyze ‘all’ metabolites, the use of internal standards certainly faces limitations due to the complexity and differences of metabolomics mixtures. Overall, metabolomics normalization has evolved in the past two decades from scaling normalizations [5,6], use of housekeeping metabolites [4], normalization based on internal or external standards [7,8,9], and quality control samples (QC)-based normalizations [10,11,12,13]; specifically, QC-based normalization methods are favored today [14]. Systematic error removal using random forest (SERRF) normalization has been shown to outperform classic QC-normalizations such as locally estimated scatterplot smoothing (LOESS) in large-scale untargeted lipidomics [13,15]. However, no such analysis has been conducted for GC–MS-based untargeted metabolomics. Interestingly, GC–MS-based metabolomics studies typically are much smaller in size than LC–MS-based studies, usually with fewer than 1500 samples [7,16,17,18,19]. Untargeted primary metabolomics on gas chromatography–mass spectrometry (GC–MS) suffers technical errors specifically due the need to increase the volatility of metabolites via chemical derivatizations. Here, we show to which extent these errors can be balanced by proper internal standards to normalize this process. In addition, involatile materials may accumulate in the GC injection liners and the beginning of the chromatography columns. Such deposits may alter the local catalysis environment for the delicate balance of derivatization products [20].

GC–MS is an ideal platform from which to detect volatile compounds. For primary metabolites with higher boiling points, a derivatization step reduces boiling points by exchanging acidic hydrogens against derivatization groups. For chemical derivatizations, a wide array of strategies and reagents can be employed, ranging from alkylations and acylations to silylations and others [21,22,23]. For example, alkylations use boron trifluoride/butanol or dimethylformamide dimethylacetals [24,25,26], silylation via *N,O*-Bis(trimethylsilyl)-trifluoroacetamide, *N*-methyltrimethylsilyl-trifluoroacetamide (MSTFA) or *N*-Methyl-*N-tert.*-butyldimethylsilyltrifluoroacetamide (MTBSTFA), acylation/esterification via propyl- or ethylchlorofomate, acetic anhydride or fluorinated anhydrides, or chiral derivatization reactions. Among silylating agents, trimethylsilylations are most frequently used in metabolomics, with MSTFA being the most widely utilized agent [27] due to its ease in handling and wide range of substrates encompassing hydroxyl-, carboxyl-, amino-, or thiol- functional groups. In contrast, other derivatization agents are hampered by less convenient operation and narrower metabolite ranges. For example, boron trifluoride in alkylation and anhydrides in acylation are corrosive, flammable, and highly toxic. 

**Figure 1:**
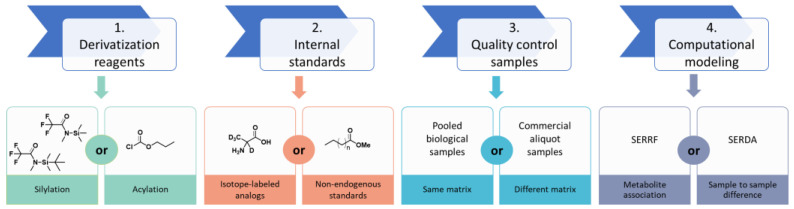
Comparisons of normalizations of large-scale GC–MS human cohort plasma datasets.

In addition, selective reagents can be used for other functional groups. We use o-methylhydroxylamine (also called methoxyamine) for 25 years in GC–MS-based metabolomics to protect carbonyl groups [28]. A range of other reagents are used in volatile analyses of flavors and odors [29]. Here, we investigate and compare different quality control strategies for metabolomics of human plasma, including derivatization agents, internal standards, external quality control samples, and computational modeling (Figure 1). In this study, we compared three derivatization agents with deuterated internal standards in three different trials across three months. We then applied two different external quality controls for a type 2 diabetes study (T2D) of >4000 human plasma samples: a QC pool made of extracts of the cohort samples and another QC pool that was commercially available. A new modeling tool, called systematic error removal using denoising autoencoder (SERDA), showed an overwhelmingly better performance than SERRF in a large-scale GC–MS-based metabolome dataset. We further compared SERDA with other traditional normalization methods (e.g., mTIC, fTIC, iTIC, metabolite–ISTD ratio) and investigated the performance by combining different normalization methods.

## 2. Materials and Methods

### 2.1. Reagents

Pooled disodium EDTA plasma was purchased from BioIVT (Westbury, NY, USA), aliquoted into portions of 30 µL, and stored at −80 °C freezer until extraction. The EZ: faast^TM^ amino acid analysis sample testing kit for propyl-chloroformate (PCF) derivatization was purchased from Phenomenex Inc. (Torrance, CA, USA). The 4104 dipotassium EDTA plasma samples were obtained from study participants of a large-scale human cohort for diabetes risk factor analysis. Samples were extracted as published previously [30,31] and aliquoted into analytical samples and backup extracts. The 1032 backup extracts were merged, homogenized, and aliquoted as QC samples. To match the cohort plasma matrix, dipotassium EDTA plasma was purchased from BioIVT (Westbury, NY, USA) and used as validation sample set.

HPLC grade extraction solvents methanol, methyl-tertiary butyl ether (MTBE), and water were obtained from Sigma-Aldrich (Dorset, UK). Twenty-five deuterium-labeled amino acids were purchased from Cambridge Isotope and were used as internal standards in the extraction solutions for the human cohort study. The following concentrations were added to plasma: alanine-d4 * (400 mM), arginine-d7 (110 mM), asparagine-d3 * (100 mM, aspartic acid-d3 * (50 mM), glutamic acid-d5 * (150 mM), glutamine-d5 * (600 mM), glycine-d5 * (400 mM), histidine-d5 (150 mM), homocysteine-d4 (100 mM), isoleucine-d10 * (100 mM), leucine-d10 * (250 mM), lysine-d8 * (200 mM), methionine-d5 * (60 mM, ornithine-d2 (100 mM), phenylalanine-d8 * (100 mM), proline-d7 (200 mM), serine-d3 * (150 mM), threonine-d5 * (200 mM), tryptophan-d8 * (80 mM), tyrosine-d7 * (100 mM), valine-d8 * (400 mM), 2-aminobutyric acid-d6 (40 mM), 2-hydroxybutyric acid-d3 (60 mM), 3-hydroxybutyric acid-d4 (100 mM), and sorbitol-d8 (50 mM). Only 16 of these amino acids (marked by asterisks) were used in the initial derivatization normalization tests of BioIVT human EDTA plasma under different reagents. 

### 2.2. Sample Preparations for GC–MS

For untargeted analyses, plasma samples were extracted using the Matyash liquid–liquid extraction method with cold methanol/MTBE/water [30]. A total of 40 µL of aliquoted plasma was thawed to room temperature and kept on ice during the following steps. Samples were vortexed for 10 s with 225 µL of ice-cold methanol, followed by adding 750 µL ice-cold MTBE. Samples were shaken for 6 min at 4 °C. A total of 188 µL of room temperature water containing the internal standards given above was added. Samples were vortexed for 20 s, followed by centrifugation at 12,210× *g* for 2 min. The lipophilic phase was decanted. The remaining hydrophilic phase was transferred to a new Eppendorf tube, dried down under vacuum and used for derivatization. 

For silylations, derivatization started using 10 μL of methoxyamine hydrochloride in pyridine (40 mg/mL, with 5 ug/mL sorbitol-d8), shaken at 30 °C for 90 min. Trimethylsilylation was performed via 90 μL N-methyl-N-(trimethylsilyl) trifluoroacetamide (MSTFA) containing C8-C30 fatty acid methyl esters (FAMEs) at 37 °C for 30 min. For derivatization with tert-butyl-dimethylsilylation, 90 μL of MTBSTFA containing C8-C30 fatty acid methyl esters (FAMEs) was used at 80 °C for 30 min. Samples were centrifuged at 12,210× *g* for 2 min and transferred to crimp top vials for GC–TOF–MS detection. 

For targeted derivatization of amino acids in 100 μL plasma sample using propyl-chloroformate (PCF), samples were prepared via a solid-phase extraction method as described previously [32], following the manufacturer’s instructions for the EZ: faast^TM^ Amino Acid Analysis sample testing kit. 

### 2.3. Gas Chromatography/Mass Spectrometry Conditions

Each mass spectrometer was coupled to an Agilent 7890 GC system (Santa Clara, CA, USA). For silylated samples, a Restek (Bellefonte, PA, USA) RTX-5Sil MS column was used (30 m length, 0.25 mm i.d, 0.25 μm df, 95% dimethyl/5%diphenyl polysiloxane film) with an additional 10 m guard column. The oven temperature was held at the initial temperature of 50 °C for 1 min, increased from 20 °C/min to 330 °C, and kept isothermal for 5 min. The injection temperature was 275 °C. The injection volume was 0.5 µL in the splitless mode. Silylated samples were measured on a LECO Pegasus IV TOF MS (St. Joseph, MI, USA) at +70 eV, source temperature 250 °C; scan range 85–700 *m*/*z* at unit resolution; sampling rate 17 Hz. 

For PCF-derivatized amino acids, a Zebron™ ZB-AAA GC column (10 m length, 0.25 mm i.d.) was used. Carrier gas (helium) flow rate was kept constant at 1.5 mL/min (60 kPa). The initial oven temperature was held at 110 °C and then ramped at 30 °C/min from 110° to 320 °C with no final hold. The injection temperature was 250 °C. The injection volume was 2.0 µL at a split ratio of 1:15. PCF-derived amino acids were analyzed by a low-resolution Agilent 5977 single quadrupole MSD (Santa Clara, CA, USA) at +70 eV, source temperature 240 °C; quadrupole temperature 180 °C; scan range 45–450 *m*/*z*, sampling rate 4 Hz.

### 2.4. Data Processing and Data Normalization Scheme

For silylated samples, raw data were deconvoluted via the Leco instrument software ChromaTOF, version 4.5. For silylated samples, deconvoluted data were submitted to the BinBase database for alignment and compound identification [20], including details on signal/noise ratios, missing peak replacements, and data curation. Data files for PCF-derived amino acids were processed using MassHunter Quantitative Analysis B.07.00 version. Data of PCF-derivatized amino acids were normalized to the corresponding internal standards, as described previously [32], and named ISTD normalization. For MTBSTFA-derivatized amino acids, the same ISTD normalization method was used according to internal standards. In addition, three sum-normalization methods were compared: (a) raw amino acid peak intensities were normalized to the sum of all deuterated internal standards, called iTIC; (b) second, data were normalized to the sum of all retention time marker compounds (fatty acid methyl esters), called fTIC; (c) third, data were normalized to the sum of all identified metabolites, as follows: amino acids, hydroxyl acids, and related compounds, called mTIC. The same methods were used to compare the normalization of trimethylsilylated samples in the initial comparison of derivatization methods. In addition, human cohort samples that underwent trimethylsilylation derivatization were normalized via two methods using quality control samples (QC): (1) SERRF (systematic error removal using random forest) [13]; and a new method that we present here: (2) SERDA (systematic error removal using denoising autoencoder; for detailed information, see the Methods section). Statistical analyses were performed via Friedman nonparametric paired tests with adjusted Dunn’s significance thresholds of *p* < 0.0332 in GraphPad Prism 8.4.3.

### 2.5. SERDA Implementation

SERDA is based on denoising autoencoder (dEA), a neural network model, and an extension of autoencoder algorithm. An autoencoder takes an input vector $x\in R^{d}$ and maps it to a hidden representation vector, $y\in R^{d^{\prime }},$ through nonlinear mapping:

y=s(Wx+b),

where s() is a nonlinear function, in our case, the element-wise exponential linear unit (elu) function; W is a $d^{\prime }\times d$ matrix; and b a $d^{\prime }\times 1$ bias vector. The hidden representation vector y is then mapped back to a reconstructed vector, $z\in R^{d},$ by

$$z=s\left(W^{\prime }y+b^{\prime }\right),$$

where $W^{\prime }$ is a $d\times d^{\prime }$ matrix; and $b^{\prime }$ a d×1 bias vector. Each element of training sample $x^{\left(i\right)}$ is mapped to a reconstruction sample $z^{\left(i\right)}$ through the hidden representation $y^{\left(i\right)}$, by minimizing the reconstruction error:

$$\frac{1}{n}\sum_{i=1}^{n}L(x^{\left(i\right)},z^{\left(i\right)}),$$

where L() is a loss function, in our case, the absolute error.

The denoising autoencoder, different from the autoencoder, first corrupts the initial input x to obtain a partially destroyed version, $\tilde{x}\in R^{d}$, by means of a stochastic mapping $\tilde{x}∽q_{D}(\tilde{x}∥x)$. In our experiments, we found that the two corruption processes—Gaussian noise and random dropout—performed satisfactorily. For the Gaussian noise, a random vector, $r\in R^{d}$, is drawn from the multivariate normal distribution, $Ν\left(0,\sigma^{2}{Ι}_{d}\right)$. For the random dropout, a fixed proportion (ν) of the components are chosen at random, and their values are forced to be 0. These corruptions not only mimic the target sample distribution but also make the algorithm less prone to overfitting. The corrupted input is $\tilde{x}=(x+r{)}_{-}$, where ()_ represents the elementwise dropout function. We chose Gaussian noise because metabolomics datasets show a normal distribution after log data transformation. The corrupted input ($\tilde{x})$ is then mapped to a hidden representation $y=s\left(Wx+b\right),$ from which we reconstruct $z=s(W^{\prime }y+b^{\prime })$. Model parameters W, b, $W^{\prime }$, and $b^{\prime }$ are trained to minimize the absolute reconstruction error using mini-batch gradient descent algorithm and backpropagation [33,34]. Supplementary Figure S1 shows a schematic representation of the process.

The construction of the SERDA algorithm can be summarized via the following steps:

(1) Take generalized log transformation on training data: $x_{1}$ (e.g., QC samples in a compound); and target data:$x_{2}$ (e.g., study samples in a compound).
(2) Draw noise from Gaussian distribution, $r∽Ν\left(0,\sigma^{2}{Ι}_{d}\right)$. Here, σ is determined by $\sigma_{2}-\sigma_{1}$, where $\sigma_{2}$ is the estimated standard deviation of $x_{2}$ and $\sigma_{1}$ is of $x_{1}$.
(3) Update training data to obtain corrupted input, ${\tilde{x}}_{1}$, by adding Gaussian noise corruption, r, to the training data (i.e., ${{\tilde{x}}_{1}=x}_{1}+r$).
(4) Optionally, oversampling n samples can be applied by adding different random Gaussian noise to each of the training data.
(5) Apply auto-scaling on the training data and target data.
(6) Split the training data, ${\tilde{x}}_{1}$, into two parts, $x_{1a}$ and $x_{1b}$, with proportions of 80% and 20%, respectively.
(7) Initialize $W\in R^{d\times d^{\prime }}$ and $W^{\prime }\in R^{d^{\prime }\times d}$ with Glorot uniform initializer. Initialize $b\in R^{d\times 1}$ and $b^{\prime }\in R^{d^{\prime }\times 1}$ by zeros.
(8) For each neural network training epoch,
  i. randomly set ν (i.e., the dropout rate) of the elements in $x_{1a}$ to zero;
  ii. randomly select b samples from $x_{1a}$ as a mini batch of samples;
  iii. update parameters W, b, $W^{\prime }$, and $b^{\prime }$ using backpropagation using the Adam algorithm [35] so that the average absolute error of the mini batch samples is reduced as much as possible;
  iv. calculate the average absolute error on $x_{1b}$ with the updated parameters.
(9) Repeat (7) i–iv until the average absolute error on $x_{1b}$ does not decrease for 50 epochs. Mark the number of epochs iteratively processed as $n_{e}$.
(10) Apply (7) i–iii on the whole training set, ${\tilde{x}}_{1}$, $n_{e}$ epochs. Denote the final trained model as $\Phi (x)=s(W^{\prime }s(Wx+b)+b^{\prime })$.
(11) Apply trained model to the target data and obtain the predicted systematic error $\Phi (x_{2})$.
(12) Calculate the normalized values, ${x_{2}}^{\prime }$, by removing the predicted systematic error with subtraction, $x_{2}-(\Phi \left(x_{2}\right)-\bar{\Phi \left(x_{2}\right)})$, where $\bar{\Phi \left(x_{2}\right)}$ is the mean average of the predicted systematic error.
(13) Optionally, median normalization can be applied to ${x_{2}}^{\prime }$ to remove leftover inter-batch effect.
(14) Scale and exponentially transform the data ${x_{2}}^{\prime }$ back to the original scale to achieve the final normalized dataset.

Hyperparameters must be provided prior to model building, including the oversampling number, n; dropout rate, ν; dimension of the hidden representation space, $d^{\prime }$; mini-batch size, b; and nonlinear function, s. These hyperparameters are determined using 5-fold cross-validation. The SERDA script is publicly available at https://github.com/slfan2013/SERDA (accessed on 11 August 2023).

### 2.6. Samples and Datasets 

For testing and validating SERDA, four types of quality control samples were used. The raw data are given in the Supplementary data file: (a) first, 413 quality control (QC) samples, pooled from a cohort of 4104 human K2EDTA plasma samples (labeled ‘pool qc’ in the Supplementary data file); (b) second, we added 409 commercial BioIVT K_2_EDTA plasma samples as independent secondary quality controls for validation purposes (labeled ‘BioIVT validation qc’ in the Supplementary data file); (c) third, 104 NIST SRM1950 human plasma QC samples [36] were added as tertiary quality controls (labeled ‘NIST validation’ in the Supplementary data file); (d) fourth, a total of 102 technical replicate samples were randomly embedded in the human cohort samples in a blinded manner. 

## 3. Results

### 3.1. GC–MS-Based Metabolomics: Data Normalization for Small Sample Sets 

**Figure 2:**
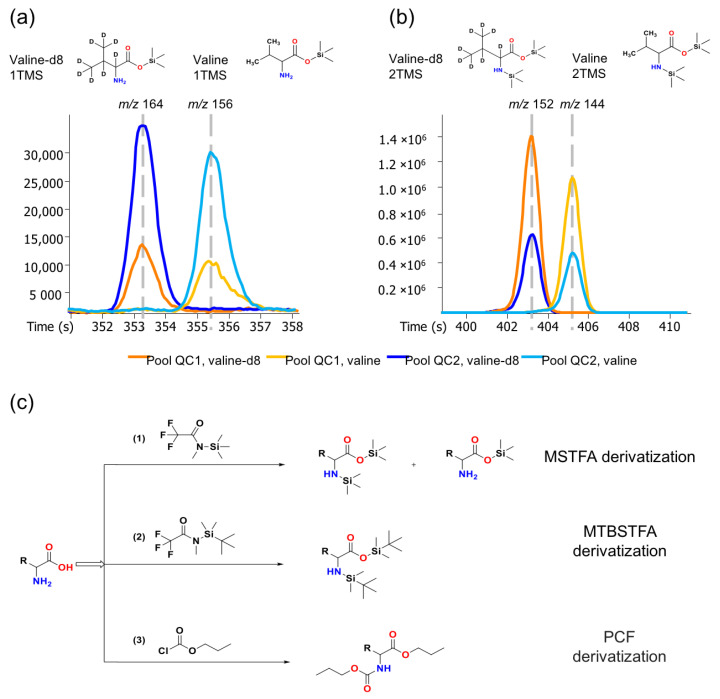
Reaction schemes of MSTFA, MTBSTFA, and PCF derivations, and the chromatography of valine derivatized with MSTFA. (**a**) valine-1TMS and valine-d8-1TMS products; (**b**) valine-2TMS and valine-d8-2TMS products; (**c**) reaction schemes for MSTFA, MTBSTFA, and PCF derivatization.

Using BioIVT human EDTA plasma, we first tested the two most common silylation reactions, trimethylsilylation (TMS) and tertiary-butyl dimethylsilylation (TBDMS), and compared these broad-range, untargeted reagents against a commercially available targeted assay for amino acid quantifications via chloroformate reaction. The broad-range silylation agents produced products with unstable ratios for primary amino groups, introducing unwanted variances in untargeted metabolomics. For example, trimethylsilylation usually generates two trimethylsilylated valine products: valine 1TMS with only the carboxyl acidic hydrogen replaced by TMS (Figure 2a, *m*/*z* 156); or valine 2TMS with one hydrogen of the amine group and the carboxyl proton replaced by TMS (Figure 2b, *m*/*z* 144). In principle, isotope-labeled internal standards should correct for such difficulties in stabilizing reaction conditions and yield exactly the same TMS–derivative ratios for amino acids if ignoring the slight chemical and physical properties between deuterium and hydrogen. We used 16 isotope-labeled metabolite analogs and spiked them into the extraction solution to correct for all technical variations as their corresponding metabolites’—from extraction to derivatization—injection to the gas chromatograph and mass spectrometry. We found that the product ratios of *N,O*-TMS-derived amino acids to only O-TMS-derived amino acids varied between pooled QC plasma samples despite all measures of pre-analytical quality controls such as regularly cutting columns, cleaning injectors, or exchanging injector needles [20]. As expected, internal stable isotope standards reduced this technical error. For instance (Figure 2a), the two trimethylsilylated products, valine-d8 1TMS (*m*/*z* 164) and valine-d8 2TMS (*m*/*z* 152), displayed similar ratios between the two QC samples as the endogenous valine 1TMS and valine 2TMS products (Figure 2a,b). 

**Figure 3:**
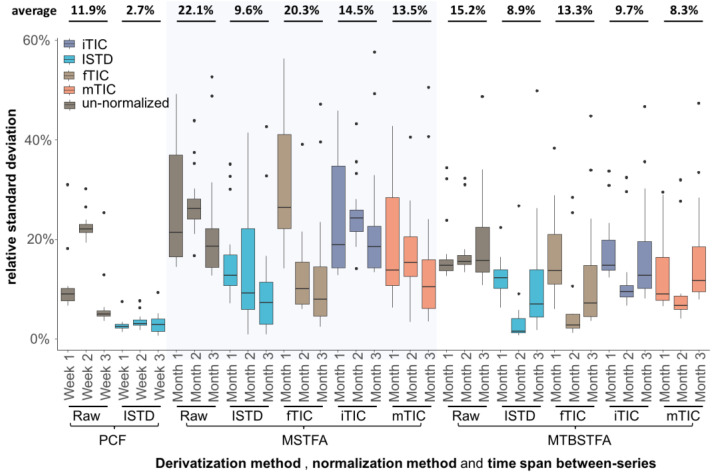
Relative standard deviation (%) of 16 amino acids by three derivatization reagents. Raw: not normalized data; ISTD: normalization of each amino acid to its corresponding internal standard; fTIC: normalization to the sum of 13 fatty acid methyl esters; iTIC: normalization to the sum of all internal isotope-labeled standards; mTIC: normalization to the sum of all identified metabolites.

However, this control for unwanted technical variation did not completely eliminate technical errors when we compared trimethylsilylation to tertiary-butyl dimethylsilylation and targeted chloroformate derivatization (Figure 2c). For this initial comparison, we used 30 commercial plasma samples that were extracted in three independent replicate studies, each conducted one month apart (Figure 3, Supplementary Table S1). We limited the analysis to 16 amino acids that were detectable in all three derivatization methods. For example, arginine was not amenable to any of the methods, while MSTFA did not yield detectable signals for histidine and cysteine in the plasma samples analyzed due to lower sensitivity. Before normalization, the raw data of all three derivatization methods showed significant variance between the three independent analyses (Figure 3). However, average raw data precision worsened from PCF to MTBSTFA to MSTFA, possibly due to the removal of matrix effects when using PCF derivatization under the Ez:faast protocol, which uses a solid-phase extraction method. Even after normalization to each individual stable-isotope-labeled amino acid, PCF derivatizations gave the lowest precision with 2.7% average relative standard deviation (RSD), followed by MTBSTFA at 8.9% RSD and MSTFA at 9.6% RSD. Although using internal standards for normalization reduced the overall systematic errors for the three tested derivatization agents, residual variance was found across all amino acids, likely due to a random combination of all analytical errors, ranging from pipetting to extraction, moisture during derivatization, and instrument performance.

In metabolomics, such precision values are regarded as acceptable. However, metabolomics aims at analyzing a wide range of compounds, with the coverage of compound classes decreasing from MSTFA to MTBSTFA to PCF derivatization. One cannot include internal standards for all possible small molecule identifications in GC–MS-based metabolomics; therefore, we tested this dataset to see whether other normalization methods might yield acceptable results for MSTFA or MTBSTFA derivatization. To this end, we used three sum-based normalizations: (a) the sum of all internal isotope-labeled standards (total ion chromatogram, iTIC); (b) the sum of all 13 fatty acid methyl esters that are added as retention index markers in our protocol (fTIC); and (c) the sum of all identified metabolites (mTIC) (Supplementary Table S1). Interestingly, the mTIC normalization worked better than iTIC or fTIC for correcting errors for the 16 amino acids for both methods, with 13.5% RSD for MSTFA and 8.3% RSD for MTBSTFA (Figure 3). Both derivatization methods showed little improvement in precision when using fTIC normalization in comparison to the raw data, possibly because fatty acid methyl esters did not undergo any derivatization but only account for random errors during injection. In comparison, iTIC normalizations yielded slightly better precisions for both MSTFA and MTBSTFA than fTIC because the individual amino acids showed similar error trend as all amino acids as a group. Nevertheless, mTIC should be regarded as the best sum-normalization method for untargeted GC–MS analyses for small datasets such as this, especially for MTBSTFA. Additionally, linearity of instrument responses may not be given for low- and-high abundant peaks, adding complexities to comparisons of RSDs across different derivatization agents.

### 3.2. GC–MS-Based Metabolomics: Data Normalization for very Large Sample Sets

**Figure 4:**
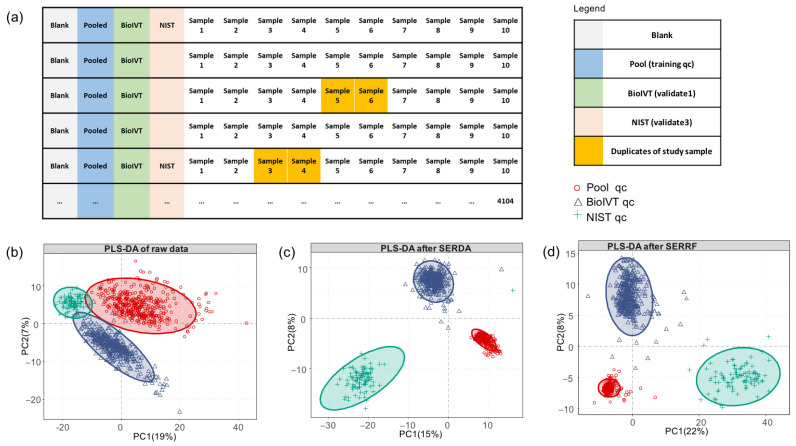
Sequence of sample acquisition and distribution of three sets of quality controls (QC) in large scale GC–MS metabolomics. (**a**) Sequence of injections of blanks, pooled sample quality controls, and BioIVT and NIST external plasma quality controls, plus blinded sample doublets. (**b**–**d**) Partial least square-discriminant analysis plots (PLS-DA) of (**b**) raw data, and effect of normalization by (**c**) SERDA and (**d**) SERRF normalization.

Most published GC–MS-based metabolomics studies use fewer than 100 samples. Only a single study has been published with almost 1200 samples [16],using relatively matrix-poor tobacco leaf extracts. Apart from instrument drifts, differences in the types and amounts of involatile residues in biological matrices (such as complex lipids or incomplete removal of proteins) may cause additional technical errors in GC–MS-based metabolomics. Here, we used human K_2_EDTA plasma samples as an example of a matrix that is highly enriched in fat and protein contents. Such plasma samples are most often used in very large clinical and epidemiological cohort studies, which makes this sample type very relevant with respect to residual technical errors. We used 25 stable-isotope-labeled metabolites during the extraction to investigate if such classic internal standards could be used beyond their corresponding unlabeled endogenous metabolites to correct for drifts during data acquisition and reduce technical (random) errors for the metabolome at large. In addition, we employed four further types of quality control samples to improve analytical precision: (a) From a cohort of 4104 human K_2_EDTA plasma samples, we pooled half of the extracts obtained from the first 1032 study samples and aliquoted this pool into 413 cohort-derived quality control (QC) samples. These QC samples were used for data normalization for MSTFA-derivatization-based GC–TOF–MS metabolomics that possess the widest range of metabolite coverage, including amino acids, bioorganic acids, sugars, hydroxyl acids, and fatty acids. Pool QC samples were added after each subset of 10 clinical cohort samples. (b) Secondly, we added one method blank and one commercial BioIVT K_2_EDTA plasma sample as independent secondary quality controls for validation purposes. (c) Thirdly, NIST SRM1950 human plasma QC samples [30,31,32,33,34] were added after each set of 40 human cohort samples. (d) Fourthly, we further analyzed a total of 102 technical replicate samples that were used within a single set of 80 samples (Figure 4a).

Data acquisition was conducted across 1.2 years in seven batches with many column cuts and >60 injection liner exchanges in addition to eight column changes and instrument autotunings following the detailed recommendations published earlier [20]. Due to these frequent but necessary interventions, raw pooled QC data showed large technical variations. We first tested the three sum-normalization methods used in the small amino-acid derivatization method sets above (fTIC, mTIC, and iTIC). As expected, none of the classic sum-based normalization methods yielded acceptable precisions for such large-scale studies, with unacceptably high median technical errors between 53–63% for both the cohort pool QC and the commercial plasma QC samples (Table 1).

Next, we used a machine-learning-based data normalization method that we previously successfully used for large-scale lipidomics data with more than 5000 samples (systematic error removal by random forest, SERRF) [13]. To avoid overfitting, we applied a five-fold cross-validation on the training QC data to calculate the RSD scores for each compound. SERRF uses correlation patterns of signal drifts of multiple metabolites in QC samples to determine correction factors that are then applied to the biological samples. SERRF avoids overcorrection by using any single metabolite, unlike the classic LOESS algorithm (locally estimated scatterplot smoothing). [37] When applied to GC–TOF–MS cohort pool QC samples, SERRF indeed greatly reduced the median technical error to only 19% RSD (Table 1), clearly below the margin of 30% RSD that had been proposed for metabolomics [38]. Correspondingly, supervised classification of the cohort pool QC, commercial pool QC, and NIST plasma pool QCs showed a large shrinkage of the data dispersion for the training data (cohort pool QC) compared to the raw data (Figure 4b,d). However, when the SERRF model was applied to the primary validation BioIVT commercial plasma QC samples, data still showed considerable dispersion (Figure 4d) and a median 34% RSD (Table 1). In comparison to the success of SERRF in lipidomics, this diminished normalization power in GC–TOF–MS metabolomics may be due to a higher random effect on absolute intensities (trimethylsilylation ratios, Figure 2a,b). In contrast, no chemical derivatization is required in lipidomics; therefore, it only has to be corrected for signal drift patterns due to systematic errors that occur gradually across hundreds of samples in a continuous way.

To better correct for random effects that may be caused by derivatizations or the less controllable splitless injection procedure in gas chromatography, we developed and applied a new normalization method, systematic error removal by denoising autoencoder (SERDA). Denoising autoencoders are used to recognize signals despite large but random noise. Denoising autoencoders randomly hide some features from input data and automatically generate a new dataset that is similar to the input data [39]. After capturing the useful information interrupted by noise signals via iterative neural network machine learning, the tool generates a reconstructed output with the same shape as the input data in the decoder stage. The denoising autoencoder method (dAE) has the following advantages when applied to data normalization: (1) it fits the structure of metabolomics data where the number of compounds is comparable to or greater than the number of samples; (2) dAE employs a nonlinear model, providing the flexibility of summarizing complex trends of systematic errors in metabolomics data; (3) dAE can tolerate multicollinearity and a high correlation across multiple metabolites, as is frequently observed in metabolomics data [15]; and (4) dAE, coupled with dropout techniques, is robust with respect to outliers and less prone to overfitting [33]. SERDA and SERRF share some similarities, such as nonlinearity and robustness. However, SERRF normalizes each compound sequentially by building a random forest model using correlations to other metabolites in the datasets, while SERDA directly predicts the pattern of systematic error for each biological sample. We can treat SERRF as a compound-by-compound normalization method, while SERDA is sample-by-sample. In LC–MS-based lipidomics, many lipids share strong correlations within each lipid class. Hence, SERRF can successfully reduce the systematic error to 5% RSD [13]. However, in GC–MS metabolomics, correlations of metabolite intensities are not only dependent on classes of chemical structures; they are also based on the stability of derivatization products and injector discrimination based on boiling points. Hence, correlations are expected to be weaker than in LC–MS, causing SERRF to be less powerful, reducing systematic errors significantly. SERDA, on the other hand, does not rely on compound correlation. It captures the data pattern of all metabolites reported by the quality control samples and predicts what the systematic error would be if a QC sample was injected in the sample position. Consequently, we found that SERDA achieved a better performance in untargeted GC–MS metabolomics datasets that show high random variance in addition to systematic drifts. For example, for training cohort QC samples in this study, a residual median technical error of 16% RSD was achieved (Table 1) along with low dispersion in multivariate clustering of the three types of QC samples (Figure 4c). Even more importantly, when the SERDA model was applied to the independent commercial BioIVT QC samples, the data dispersion in the multivariate cluster was smaller than with the SERRF data (Figure 4c,d), and the overall median errors after SERDA modeling were found at 17% RSD, well within the acceptable limits in untargeted metabolomics. Although SERDA requires cross-validation for hyperparameter tuning, we observed that the performance of SERDA was robust against hyperparameter selection. All combinations achieved a consistently lower RSD than SERRF. In addition, when we tested the effect of sum-normalization on of SERDA modeled data, neither mTIC, fTIC, nor iTIC normalizations further improved the residual technical errors of the SERDA data (Table 1).

**Figure 5:**
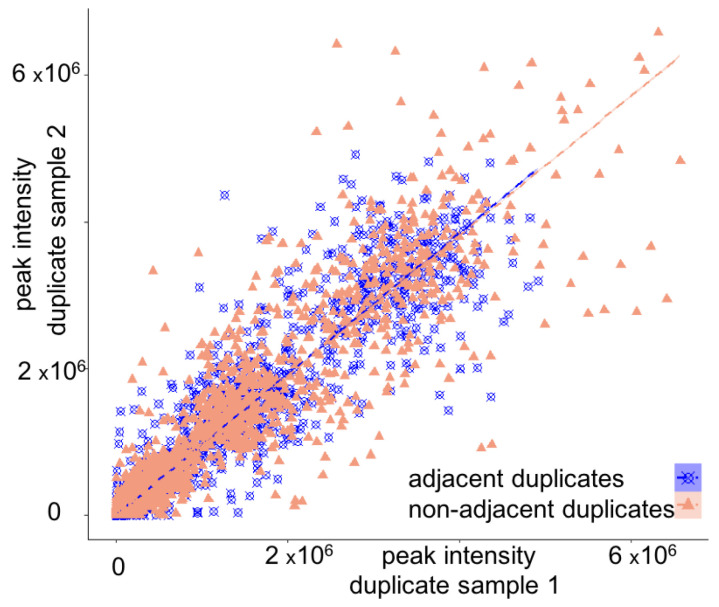
Correlation of blinded T2D cohort sample duplicates after SERDA normalization.

Next, we analyzed the effect of SERDA on 102 biological duplicate samples that were interspersed into the data acquisition of the 4104 cohort study samples (Figure 5, Supplementary Table S2). A total of 48 biological replicates were measured in adjacent positions within GC autosamplers (Figure 4a), while 54 additional biological replicates were measured apart within a set of 80 samples. Across all metabolites, correlation coefficients for both adjacent and nonadjacent biological replicates were found at excellent *r*_xy_ = 0.98, with ranges of *r*_xy_ = 0.81 to 1.0 for nonadjacent replicates that were only slightly worse than adjacent replicates with ranges of *r*_xy_ = 0.90 to 1.0 (Figure 5, Supplementary Table S2). These data showed that the SERDA algorithm correctly normalized metabolite intensities in biological replicates for both high- and low-abundant compounds.

**Figure 6:**
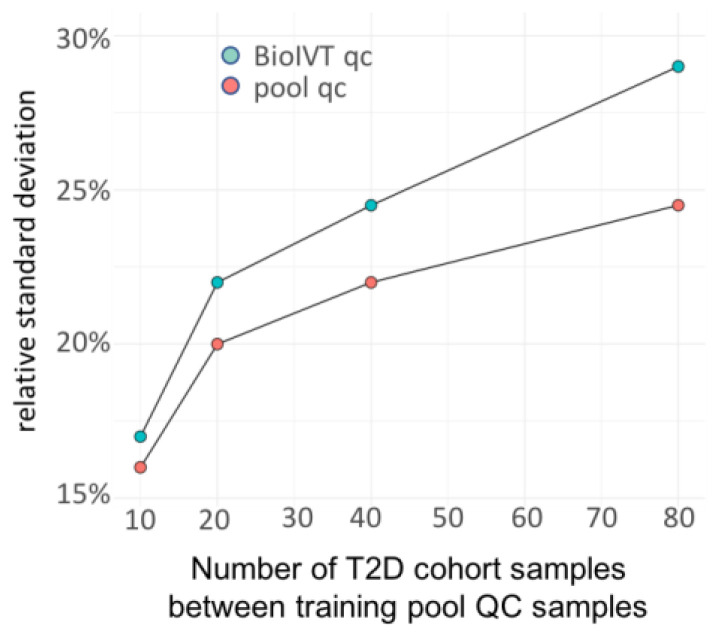
The relative standard deviation (RSD) of pool QC samples (red) and BioIVT qc samples (blue) for each set of 10, 20, 40, and 80 biological samples. With a smaller number of training QC samples, the performance of SERDA decreases, as indicated by the increasing of both the RSD of pool QC traing qc samples and BioIVT validation qc samples.

We then investigated how the number of training cohort QC samples affected the overall efficiency of the SERDA algorithm. To this end, we used fewer cohort QC samples for training and then applied the SERDA models on the remaining cohort QC pool samples and the commercial BioIVT QC pools. Median technical errors for commercial BioIVT pools worsened from 17% RSD when using training QC samples after each set of 10 biological samples to 20%, 22%, and 25% RSD when using training QC samples after each set of 20, 40, or 80 biological samples (Figure 6). Technical errors for the remaining cohort QC pool followed the same trend when using SERDA models for each set of 20, 40, or 80 biological samples (Figure 6). Interestingly, technical errors for cohort QC pool samples remained 1–5% RSD lower when used as testing samples than errors obtained via the BioIVT commercial pool samples, and the longer the pooled QC interval, the larger the difference. This observation can be explained by the slight differences in biological matrix compositions between the plasma QC pool of a specific age and ethnicity composition of a human cohort study, as well as the composition of commercial BioIVT samples. Based on these data, we recommend using one cohort pool QC sample for every 10 biological samples as it yielded significantly better precision than using fewer cohort QC pool samples. We also advocate for using secondary-test QC samples—as shown here by commercial BioIVT QC plasma samples—to independently test for the overall effect of the SERDA models.

**Figure 7:**
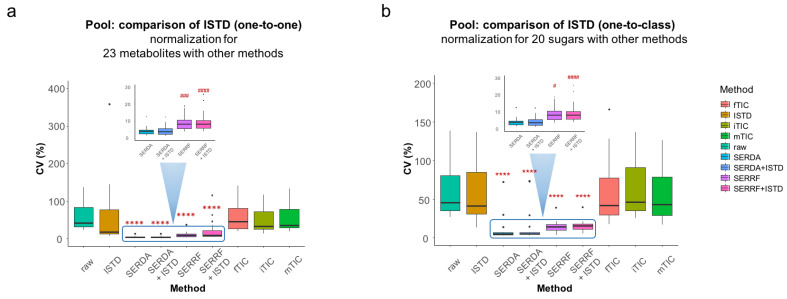
Comparison of ISTD absolute ratio normalization with QC-based and TIC-based normalization methods. The Friedman nonparametric test was used for significance comparison with raw. *p*-value threshold: 0.1234 (ns), 0.0001 (****). The Friedman nonparametric test was used for significance testing compared to SERDA. *P* value threshold: 0.1234 (ns), 0.0332 (#), 0.0002 (###), 0.0001 (####). (**a**) One-to-one: the absolute ratio was calculated by dividing the peak intensity of endogenous metabolite by the corresponding deuterated ISTD; (**b**) One-to-class: the absolute ratio was calculated by dividing the peak intensity of endogenous metabolite by a single deuterated compound as an analog ISTD for the entire class.

Furthermore, we investigated how SERDA normalization compared to the use of 25 internal standards (ISTD) that were spiked into the plasma extraction solution. For 23 of these compounds, the corresponding unlabeled endogenous plasma metabolites levels were detected by GC–TOF–MS metabolomics; two additional internal standards (homocysteine-d4 and sorbitol-d8) were structurally similar to endogenous metabolites (cysteine and blood sugar alcohols). When using the 23 ISTDs to normalize, one-to-one, to their endogenous plasma counterparts, a median 19% RSD was found, with proline and histidine as outliers (Figure 7a). For the same 23 endogenous compounds, SERDA normalization achieved a much lower technical error of 3.8% RSD, and even SERRF outperformed the use the internal standards, with 5% RSD (Figure 7a). The same trends were observed for both cohort pool QC samples and commercial BioIVT plasma QC samples (Figure 7a and Supplementary Figure S2). Use of sum-normalization methods (mTIC, fTIC, iTIC) worsened the performance of the SERRF or SERDA methods alone and did not outperform the one-on-one use of internal standards either (Figure 7a). When we used sorbitol-d8 as the sole internal standard surrogate for a class of 20 detected sugars, sugar alcohols, or disaccharide, this ‘one-to-class’ normalization failed to improve the technical errors for these 20 sugars, whereas SERDA still outperformed SERRF with respect to carbohydrates for both cohort pool QCs and commercial BioIVT QCs (Figure 7b and Supplementary Figure S2).

Last, we compared the performance of the SERDA method against batchwise-LOESS and SERRF on three GC–MS datasets with respect to the outcome of error residuals using the median RSDs across all compounds [40]. A detailed description of the metabolomics studies can be found in the Supplementary Table S3.

**Figure 8:**
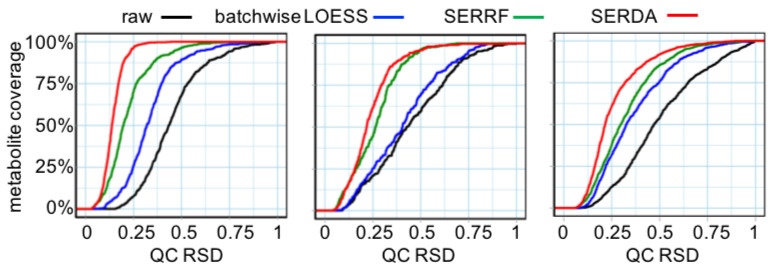
Effect of different normalization methods on residual errors in GC–MS-based metabolomics datasets. Left panel: this study, N = 413 quality control human plasma samples (QC), reporting 661 metabolites. Mid panel: 104 human plasma QC samples of the GeneBank study on 319 metabolites. Right panel: 30 QC plasma samples of the MPA study on 991 metabolites. For each panel, cumulative distributions of cross-validated relative standard deviations (RSD) are given using raw (black), batchwise-LOESS (blue), SERRF (green), and SERDA (red) normalized dataset. The coverage of metabolites achieving specific RSD levels is given as the *y*-axis.

To avoid overfitting, we applied a five-fold cross-validation on the training QCs data to calculate the RSD scores for each compound. For the current T2D study QCs, we added external validation data by employing commercial BioIVT human plasma samples. Such samples were not available for the GeneBank study because at that time, secondary pool QCs were not deemed necessary, and certainly not for the MPA study because the MPA study compared different animals. A successful normalization method should yield small RSDs for both cross-validated training QCs and external-validation QCs. In addition, pool quality controls that were left out of model training provided an excellent opportunity to evaluate the performance of all models without the risk of overfitting, in addition to the external validation BioIVT plasma QCs. Table 2a,b shows that SERDA normalized datasets achieved significantly lower cross- and external-validation RSD compared with SERRF normalization (Wilcoxon signed-rank test, *p*-value = 0.02). Residual errors were reduced by SERDA by an additional 3–6% RSD in cross-validated training data compared to SERRF normalization for the three studies, and by more than the additional 17% RSD for the external BioIVT plasma QCs. Such error reduction meant that for the three datasets, the coverage of compounds largely increased that achieved <30% RSD, a typical threshold used for metabolomic datasets [38] (Figure 8).

SERDA yielded the highest coverage of compounds with acceptable residual errors for the three GC–MS datasets, ranging from 72–98% of all metabolites (Table 3). In comparison, SERRF only achieved a coverage of 50–81% of all metabolites reaching that precision threshold (Table 3). Details of the precision coverage for each method is given in Figure 7, which shows the cumulative distribution of the cross-validated RSDs of the quality controls for the raw data and the batchwise-LOESS, SERRF, and SERDA normalized data. SERDA consistently reached a higher portion of smaller RSDs across all the compounds compared with the other two methods. The T2D study was much larger in size compared to the GeneBank and MPA studies, leading to a much larger number of quality control samples employed in the normalization. Hence, difference in improvement of residual errors by the SERDA method may be explained by the number of QCs in the datasets. This finding is consistent with the results of our investigation into using fewer QC samples in the T2D study (Table 2), where the performance of SERDA was found to be increasingly improved with a larger number of QC samples. We conclude that SERDA works well for large-scale GC–MS normalization, while it can still achieve considerable improvement in small-sample settings.

## 4. Conclusions

In summary, we present the results of different normalization methods for GC–MS-based metabolomics studies in human plasma. While MSTFA derivatization showed higher technical errors for amino acids than alternative MTBSTFA or PCF derivatizations, due to the much broader coverage of plasma metabolite annotations for trimethylsilylated compounds, MSTFA is still the reagent of choice for GC–MS profiling. Yet, due to both random and systematic errors in large-scale human plasma cohort studies, the technical errors in GC–MS-based screening of primary (polar) metabolites are harder to control than in LC–MS-based lipidomics [13]. Neither classic internal standards nor sum-based normalizations were sufficient to correct for GC–MS drifts, and even the machine-learning-based SERRF method did not yield satisfying results. However, using denoising autoencoder algorithms implemented in the SERDA random neural networks presented here reduced the median residual errors to less than 20% RSD, making the data usable for epidemiological statistics. This human plasma dataset was acquired over 1.2 years. Date/time stamps in the supplemental dataset showed that effects of breaks in the data acquisition schedule, e.g., for maintenance, were overcome by the SERDA algorithm. To match blood matrix effects in the best way possible, we strongly recommend using quality control samples pooled from the exact same samples as the human cohort study. Commercial plasma QC or NIST SRM1950 QC samples should only be used as independent test samples to estimate the overall residual technical errors. The use of internal standards should be limited to estimating the absolute concentrations of specific plasma metabolites; it should not be used for broad-scale normalization schemes.

## Figures and Tables

**Table 1:** Comparison of relative standard deviations (%RSD) of different normalization methods using pooled T2D plasma extracts (after every 10th sample) as the training QC set and BioIVT as the validation QC set versus normalizations to sum intensities of FAMEs (fTIC), internal standards (iTIC), or all identified metabolites (mTIC).

	SERDA			SERRF			fTIC		iTIC		mTIC		Raw	
	Pool QC	Cross-Valid.	BioIVT Valid.	Pool QC	Cross-Valid.	BioIVT Valid.	Pool QC	BioIVT Valid.	Pool QC	BioIVT Valid.	Pool QC	BioIVT Valid.	Pool QC	BioIVT Valid.
Median	5%	16%	19%	13%	19%	34%	53%	51%	59%	63%	53%	60%	58%	56%
Mean	15%	25%	24%	15%	21%	53%	74%	67%	83%	80%	75%	83%	83%	74%

**Table 2:** Median % relative standard deviation (RSD) of raw data compared to cross-validated % RSD of SERRF and SERDA normalized data. Median % RSD of raw data compared to externally validated %RSD of SERRF and SERDA normalized data using BioIVT plasma quality control samples.

GC–MS Study	Raw Data	SERRF	SERDA
GeneBank	55%	25%	21%
T2D	58%	19%	16%
MPA	50%	28%	22%
GC–MS study	raw data	SERRF	SERDA
T2D	56%	34%	17%

**Table 3:** Percentage of compounds with cross-validated relative standard deviation < 30% (GC–MS) for raw data, SERRF, and SERDA normalized data. Percentage of compounds with externally validated relative standard. deviation < 30% (GC–MS) for raw data, SERRF, and SERDA normalized data.

Dataset	Raw Data	SERRF	SERDA
GeneBank	27%	61%	76%
T2D	15%	81%	98%
MPA	17%	50%	72%
dataset	raw data	SERRF	SERDA
T2D	12%	67%	91%

## Data Availability

Processed, un-normalized data of the pool quality controls for the large T2D human cohort study are available for download as a supplementary data file. Raw unprocessed data files can be requested from OF.

## Supplementary Materials

The following supporting information can be downloaded at: https://www.mdpi.com/article/10.3390/metabo13080944/s1, Supplementary Data File: raw data output from GC-BinBase data processing of Type 2 diabetes cohort samples and pooled QC, BioIVT qc and NIST QC samples. Table S1: Average of the median relative standard deviation of 16 amino acids by three derivatization reagents across three months; Table S2: Correlation coefficients rxy of biological duplicates after normalization by SERDA; Figure S1: A schematic representation of a denoising autoencoder algorithm; Figure S2: Comparison of absolute ratio normalization using internal standards (ISTD) with QC-based and sum-based normalization methods; Table S3: Overview of metabolomics studies used for development and validation of the SERDA algorithm.
