# Supplementary material for: Denoising Autoencoder Normalization for Large-Scale Untargeted Metabolomics by Gas Chromatography–Mass Spectrometry
Source: Metabolites. 2023 Aug 13;13(8):944. doi: 10.3390/metabo13080944 (PMC10456436; doi:10.3390/metabo13080944)
Supplement: Supplementary file 1 [file metabolites-13-00944-s001.zip › Supplementary Information_Ying Zhang_Sili Fan_et al_Metabolites_08-2023.pdf]

# *Supplementary Information* for

# Denoising autoencoder normalization for large-scale untargeted metabolomics by gas chromatography-mass spectrometry

**Ying Zhang[1], Sili Fan[1], Gert Wohlgemuth[1] and Oliver Fiehn [1,*]**
[1] **West Coast Metabolomics Center, UC Davis**
* Correspondence: ofiehn@ucdavis.edu

Supplementary Figure S1. A schematic representation of a denoising autoencoder algorithm.

Supplementary Figure S2. Comparison of absolute ratio normalization using internal standards (ISTD) with QC-based and sum-based normalization methods

Supplementary Table S1. Average of the median relative standard deviation of 16 amino acids by three derivatization reagents across three months

Supplementary Table S2. Correlation coefficients $r_{xy}$ of biological duplicates after normalization by SERDA.

Supplementary Table S3. Overview of metabolomics studies used for development and validation of the SERDA algorithm

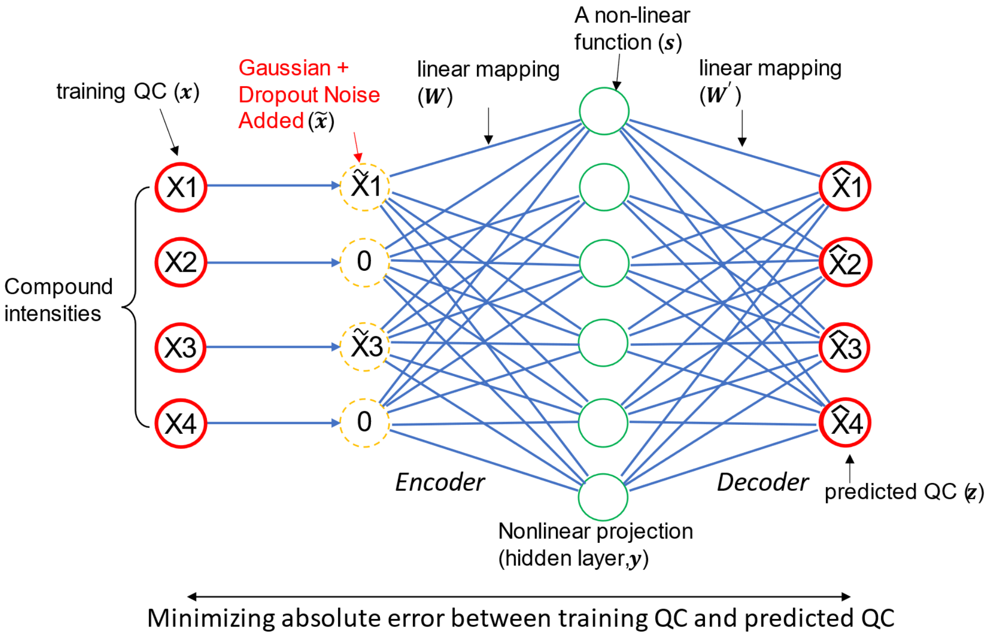


**Supplementary Figure S1.** A schematic representation of a denoising autoencoder algorithm.

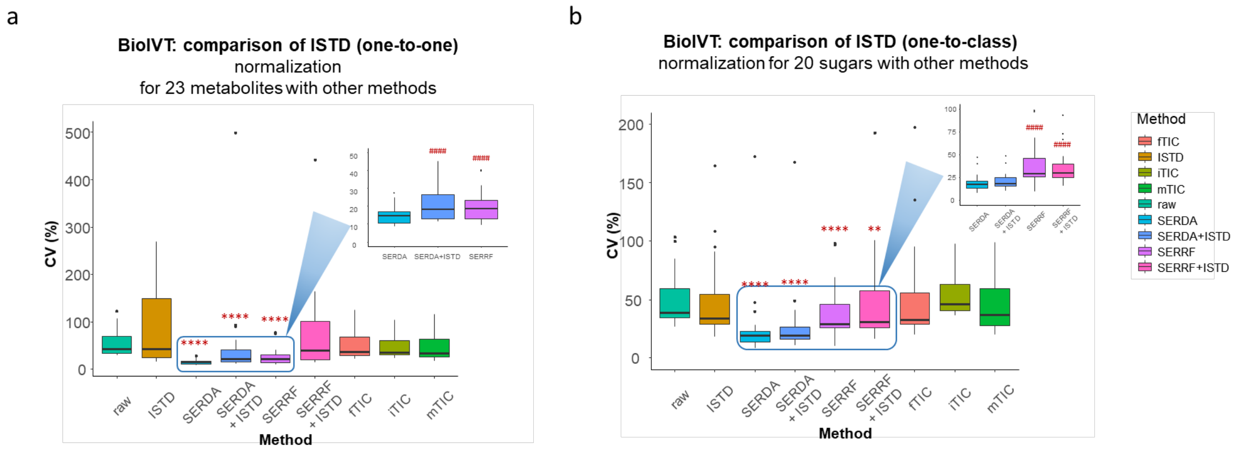


**Supplementary Figure S2.** Comparison of absolute ratio normalization using internal standards (ISTD) with QC-based and sum-based normalization methods. The Friedman nonparametric test was used for significance comparisons. P value threshold: 0.1234 (ns), 0.0332 (#), 0.0002(###), 0.0001(####). (a) One-to-one: the absolute ratio was calculated by dividing the peak intensity of endogenous metabolite by the corresponding deuterated ISTD; (b) One-to-class: the absolute ratio was calculated by dividing the peak intensity of endogenous me-tabolite by a single deuterated compound as an analog ISTD for the entire class.

**Supplementary Table S1**. Average of the median relative standard deviation (%RSD) of 16 amino acids detected in BioIVT human EDTA plasma using three derivatization reagents across three months: alanine, asparagine, aspartic acid, glutamic acid, glutamine, glycine, isoleucine, leucine, lysine, methionine, phenylalanine, serine, threonine, tryptophan, tyrosine, valine (including their corresponding deuterated internal standards).

| Reagents | Raw | ISTD | fTIC | iTIC | mTIC |
|---|---|---|---|---|---|
| **PCF** | 11.9% | 2.7% | | | |
| **MSTFA** | 22.1% | 9.6% | 20.3% | 14.5% | 13.5% |
| **MTBSTFA** | 15.2% | 8.9% | 13.3% | 9.7% | 8.3% |

**Supplementary Table S2**. Correlation coefficients $r_{xy}$ of biological duplicates after normalization by SERDA.

| Statistics for correlation | **# duplicates** | $r_{xy}$ **average** | $r_{xy}$ **standard deviation** | $r_{xy}$ **max.** | $r_{xy}$ **min.** |
|---|---|---|---|---|---|
| run order adjacent biological duplicates | 48 | 0.98 | 2.15% | 1.00 | 0.90 |
| run order non-adjacent biological duplicates | 54 | 0.98 | 3.03% | 1.00 | 0.81 |
| **Total** | 102 | 0.98 | 2.64% | 1.00 | 0.81 |

**Supplementary Table S3.**
Overview of metabolomics studies used for development and validation of the SERDA algorithm

| dataset number | Study | Type | number of study samples | number of pooled QC | number of commercial QC* | number of compounds |
|---|---|---|---|---|---|---|
| 1 | GeneBank | | 1157 | 104 | - | 319 |
| 2 | T2D | GC-MS | 4104 | 413 | 413 | 661 |
| 3 | MPA | | 300 | 30 | - | 991 |

* *Commercial QC samples are only available for T2D GC-MS datasets.*

*GeneBank study (GC-MS)*
Polar metabolites were extracted from a plasma sample (30 µL) following protein precipitation using an acetonitrile/isopropanol/water (3:3:2, v/v/v) mixture (1 mL). Three aliquots (each 300 µL) were collected. The first aliquot was used for GC-MS analysis, the second one was used for HILIC-MS and the third one served as backup.
For GC-MS, the aliquot was evaporated and resuspended using an acetonitrile/water (1:1, v/v) mixture (500 µL), vortexed, centrifuged, and 450 µL of the supernatant was collected to a new tube and evaporated. Polar metabolites were derivatized with 10 µL of methoxyamine hydrochloride in pyridine (40 mg/mL) by shaking at 30°C for 90 min followed by trimethylsilylation with 60 µL of N-methyl-N-(trimethylsilyl) trifluoroacetamide (MSTFA) by shaking at 37°C for 30 min containing C8–C30 fatty acid methyl esters (FAMEs) as internal standards. Derivatized samples were subsequently submitted for analysis by GC-MS (0.5 µL injection). Primary metabolite data was collected using a Leco Pegasus IV time-of-flight (TOF) MS (Leco Corporation) coupled to an Agilent 6890 GC (Agilent Technologies) equipped with an Rtx-5Sil MS column (30 m · 0.25 mm id · 0.25 µm film thickness, Restek) and a Gerstel MPS2 automatic liner exchange system (Gerstel GMBH & Co. KG). The chromatographic gradient used a constant flow of 1 mL/min with following gradient: 50°C (1 min),

20°C/min to 330°C, hold 5 min. Mass spectrometry data was collected at m/z 85–500 with 17 spectra/s, electron ionization at −70 eV and an ion source temperature of 250°C. Data was processed by ChromaTOF for deconvolution, peak picking, and BinBase [DOI: 10.1186/1471-2105-12-321] for metabolite identifications.

*T2D study (Sample extraction)*

Plasma samples were extracted based on modified liquid-liquid extraction method with cold methanol/ MTBE/water. 40 µL of aliquoted plasma was thawed to room temperature and kept on ice during the following steps. First, 225 µL of cold methanol with 14 internal standards(ISTD) [PC(12:0/13:0), PE(17:0/17:0), PG(17:0/17:0), LPC(17:0), LPE(17:1), d7-cholesterol, SM(d18:1/17:0), C17 Ceramide, sphingosine (d17:1), MG(17:0/0:0/0:0 ), DG(12:0/12:0/0:0), DG(18:1/2:0/0:0), and d5-TG(17:0/17:1/17:0)], d3-palmitic acid) was added to the samples followed by vortexing for 10s. Then 750 µL of cold MTBE with CE 22:1 (ISTD) was added and vortexed for 15s. The samples were shaken for 6min at 4 °C. Then, 188 µL of room temperature HPLC/MS grade water with 28 ISTD (alanine-d4, arginine-d7, asparagine-d3, aspartic acid-d3, cystine-d4, glutamic acid-d5, glycine-d5, histidine-d5, homocysteine-d4, isoleucine-d10, leucine-d10, lysine-d8, methionine-d5, ornithine-d2, phenylalanine-d8, proline-d7, serine-d3, threonine-d5, tyrosine-d7, valine-d8, glutamine-d5, tryptophan-d8, 2-keto-4-methylpentanoic acid-d3, 2-aminobutyric acid-d6, 2-ketobutyric acid-d4, 2-hydroxybutyric acid-d3, 3-hydroxybutyric acid-d4, 3-methyl-2-oxovaleric acid-d8 ) was added and the samples were vortexed for 20s. The samples were centrifuged at 12,210 × g for 2 min. 350 µL of lipophilic phase was transferred and dried down. The remaining hydrophilic phase was subjected to further treatment for polar small molecules analysis. The hydrophilic phase was then transferred and added 750 µL cold EtOH/IPA (v/v: 1/1). Samples were vortexed by 20s and centrifuged at 12,210 × g for 2 min. 450 µL supernatant was taken and dried down.

*T2D study (GC-MS-based primary metabolites assay)*

Hydrophilic parts were derivatized by adding 10 µL of methoxyamine hydrochloride in pyridine (40 mg/mL, with 5 ug/mL sorbitol-d8) and shaken at 30°C for 90 min. Then, 90 µL of N-methyl-N-(trimethylsilyl) trifluoroacetamide (MSTFA) containing C8-C30 fatty acid methyl esters (FAMEs) was added and samples were shaken at 37°C for another 30 min. 0.5 µL of derivatized sample was injected and metabolites were separated using an Agilent 7890 GC system (Santa Clara, CA) with a RTX-5Sil MS column (30m length, 0.25 mm × 0.25 µM, 95% dimethyl/5%diphenyl polysiloxane, Restek, Bellefonte, PA). The chromatographic gradient with a constant flow of 1 mL/min was listed below: 50°C (1 min), 20°C/min to 330°C, hold 5 min. Mass spectrometry parameters were used with electron ionization voltage (−70 eV), ion source temperature (250°C), mass range (85–500 m/z), and acquisition speed (17 spectra/s), Data was processed by ChromaTOF for deconvolution, peak picking, and BinBase for large scale sample size metabolite identification.

*MPA (GC-MS-based primary metabolites assay)*

Polar metabolites were extracted from plasma and serum samples (20 µL) following protein precipitation with acetonitrile/isopropanol/water (3:3:2, v/v/v) mixture (670 µL). Two aliquots (each 250 µL) were collected. The first aliquot was used for GC-MS analysis, the second one served as backup.

Aliquots were evaporated to dryness and resuspended using an acetonitrile/water (1:1, v/v) mixture (400 µL), vortexed, centrifuged, and 150 µL of the supernatant was collected to a new tube and evaporated. Polar metabolites were derivatized with 10 µL of methoxyamine hydrochloride in pyridine (40 mg/mL) by shaking at 30°C for 90 min followed by trimethylsilylation with 60 µL of N-methyl-N-(trimethylsilyl) trifluoroacetamide (MSTFA) by shaking at 37°C for 30 min containing C8–C30 fatty acid methyl esters (FAMEs) as internal standards. Derivatized samples were subsequently submitted for analysis by GC-MS (0.5 µL injection). Data was collected as described above (GeneBank study).
